# A Head-Mounted Spectacle Frame for the Study of Mouse Lens-Induced Myopia

**DOI:** 10.1155/2016/8497278

**Published:** 2016-01-19

**Authors:** Yangshun Gu, Baisheng Xu, Chunfei Feng, Yang Ni, Qin Wu, Chixin Du, Nan Hong, Peng Li, Zhihua Ding, Bo Jiang

**Affiliations:** ^1^Department of Ophthalmology, First Affiliated Hospital, College of Medicine, Zhejiang University, Hangzhou, Zhejiang 310003, China; ^2^Department of Operation Room, First Affiliated Hospital, College of Medicine, Zhejiang University, Hangzhou, Zhejiang 310003, China; ^3^State Key Lab of Modern Optical Instrumentation, Department of Optical Engineering, Zhejiang University, Hangzhou, Zhejiang 310027, China

## Abstract

The mouse model has been widely employed to explore the mysteries of myopia. For now, existing techniques for induction of experimental myopia in mice can be classified into three types: (1) devices directly glued to the fur; (2) devices attached using a combination of glue and sutures; (3) devices attached using a skull-mounted apparatus. These techniques each have its advantages, disadvantages when considering the devices stability, safety, complexity, effectiveness, and so forth. Thus, techniques for myopia induction in mice have yet to be further refined to popularize the applications. In this pilot study, we introduce a new head fixation device named the head-mounted spectacle frame apparatus for the study of mouse lens-induced myopia. Surgical procedures for device attachment were relatively simple and easy to learn in our study. Effective myopia induction was validated by retinoscopy refraction and axial length measurement using optical coherence tomography. In addition, it showed improved compliance and reliable safety when compared to the published methods. The head-mounted spectacle frame apparatus provides a new choice for the study of lens-induced myopia in mouse. It also allows for the use of form deprivation, making it attractive for future experimental mouse myopia trials.

## 1. Introduction

For the past half century, myopia has emerged as an extremely important health issue and the related literature has increased exponentially [1, 2]. And epidemiological data suggested that the prevalence and incidence of myopia were increasingly high, causing serious social and economic burdens [3]. So both scientists and ophthalmologists showed great interest and applied significant effort to elucidate the mechanism of myopia development.

As an important component in the field of myopia research, animal model has greatly expanded our knowledge on the visual regulation of refractive development [4, 5]. These animal models such as monkeys, chicks, tree shrews, fish, and guinea pigs each have its advantages and disadvantages [6–8]. However, presently consensus has not been established yet regarding which model is ideal. Recently, the establishment of mouse model of myopia has contributed to a recent surge in interest and revealed several essential findings [9–11]. The mouse model offers a highly efficient model in which to study the genetic and environmental basis of the growth of the eye, as well as gene-environment interactions [12–14]. Nevertheless, available techniques for device attachment in mice are limited. In methods using sutures, glue, or a combination of techniques, the goggle or defocusing lens may be easily scratched off and lead to poor ocular health [11, 15, 16]. Other researchers introduced an Elizabethan collar to avoid troublesome scratches, but the collar may seriously impact the animal's activity and systemic health [11, 17]. The head-mounted goggle apparatus was reported to be highly stable and efficient. However, the components of goggle apparatus were not convenient fabrication and the surgical procedures were complicated [18].

Then, the purpose of this study is to introduce a simple head fixation device for myopia induction in mice that (1) can be attached with simplified procedures, (2) should keep stable for a certain period of time, (3) effectively induce experimental myopia, and (4) is reliably secured to the eye and whole health.

## 2. Materials and Methods

### 2.1. Design of the Head Fixation Device

**Figure 1:**
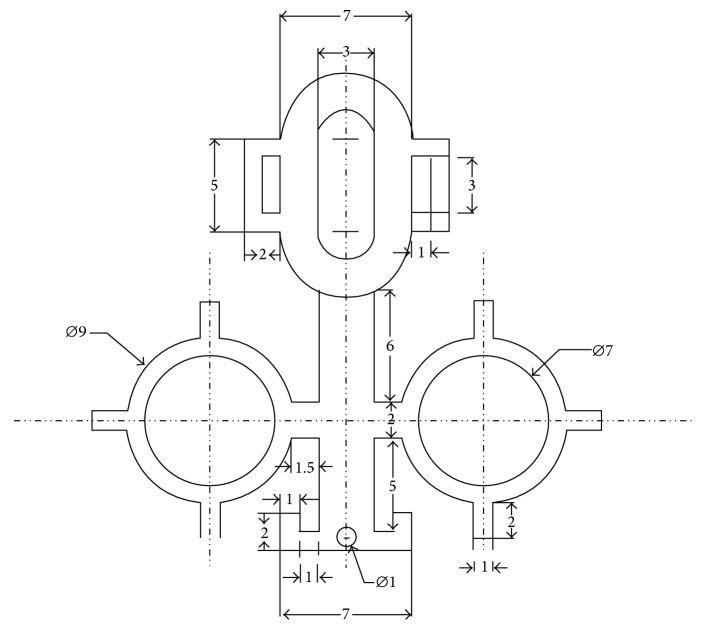
The physical dimensions for the frame (unit: mm).

**Figure 2:**
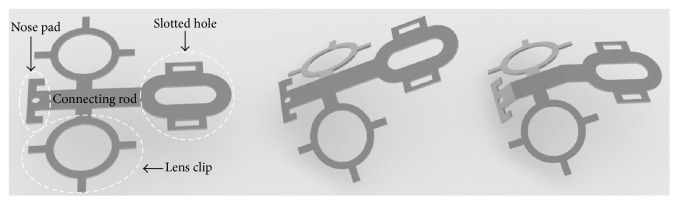
A schematic of the spectacle frame showing the functional components including the slotted hole, lens clip, nose pad, and connecting rod. And a brief visualization of the 3D shape is present.

The head fixation device is composed of two parts, the nylon connector and the metal spectacle frame. The nylon connector, including M3 × 8 nylon screws, gaskets, and caps, was designed and produced (Shanghai Zhisheng Plastic Co., Ltd.). The spectacle frames were first designed using Rhinoceros software (Version 5.0, Robert McNeel & Associates; Figure 1) and then were made of 304 stainless steel by laser cutting (Versolsolar Hangzhou Co., Ltd.). A single spectacle frame has four functional components including the (1) kidney-shaped slotted hole for position control, (2) rounded lens clip for experimental lens fixation, (3) nose pad for strengthening fixation, and (4) connecting rod for integration. The spectacle frames underwent manual shaping to be attached to the mouse head; the physical dimensions and a computer representation of the 3D shape for the frame are shown in Figures 1 and 2.

### 2.2. Surgical Procedures

**Figure 3:**
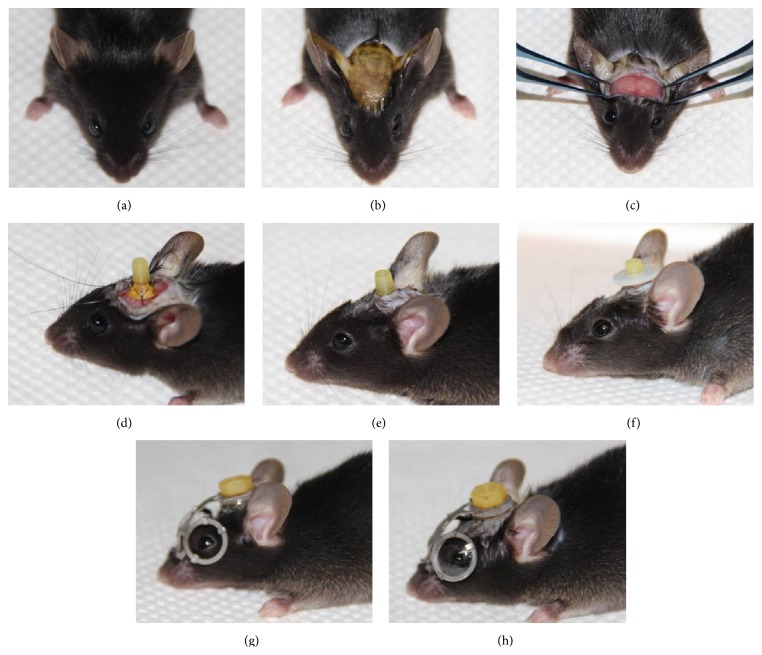
Simplified operation procedures of the head-mounted spectacle frame apparatus: (a) general anesthesia; (b) incision preparation; (c) exposure of the skull and muscle; (d) suturing the screw; (e) suturing the incision; (f) fixing the gasket; (g) fixing the spectacle frame; and (h) fixing the experimental lenses.

1. The mice were anesthetized by intraperitoneal injection of 4% chloral hydrate (10 mg/kg, Figure 3(a)).
2. The dorsal cranial fur was shaven; the surgical area was cleaned with Betadine (Figure 3(b)).
3. A middle line incision (8–10 mm) was made to expose the dorsal cranial surface of the skull.
4. The exposed fascia and periosteum were removed from the coronal suture to the sagittal suture, and the surface was cleaned and dried (Figure 3(c)).
5. The screw was sutured to the bilateral temporal muscle, cervical trapezius muscle, and frontal skin with 6-0 nylon sutures (Figure 3(d)).
6. The incision was sutured with 6-0 nylon suture, and antibiotic ointments were applied (Figure 3(e)).
7. The gasket and screw were fixed to supply a platform supporting the spectacle frame (Figure 3(f)).
8. The spectacle frame was placed on the head, and the cap was screwed in and adjusted to position the lens clip (Figure 3(g)).
9. The fixation was strengthened by suturing the nose pad to the skin, and the experimental lenses were fixed (Figure 3(h)).

### 2.3. Experimental Design

**Figure 4:**
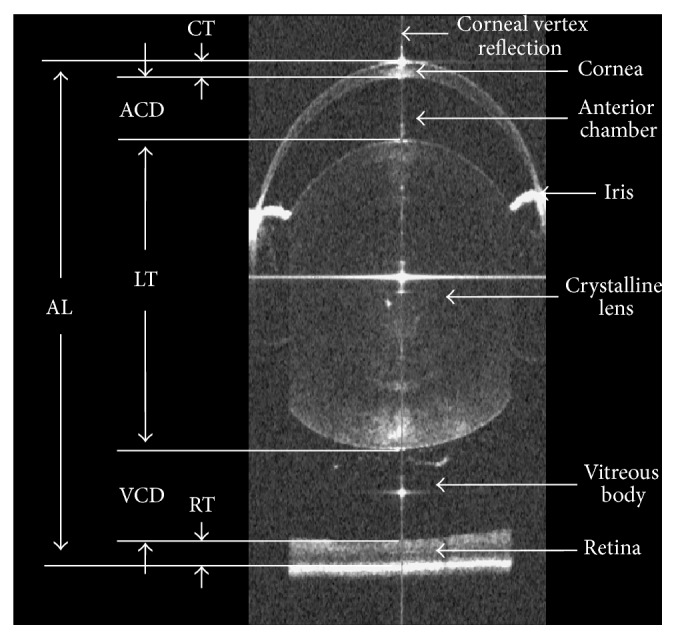
The schematic diagram of axial length measurement from the anterior corneal surface to the retinal pigment epithelium along the corneal vertex reflection.

Male C57BL6J mice aged postnatal 28 days (P28) underwent baseline retinoscopy refraction and axial length measurement using a custom-built optical coherence tomography (OCT), which has been detailed in [19, 20] (Figure 4). Two groups were set in the current study. In the experimental group (*n* = 25), myopia was monocularly induced with the head-mounted spectacle frame over the right eye using a −15.0 diopter (D) lens (Hangzhou Boston Optics Co., Ltd.); no defocusing lens or plano lens was fixed to the left lens clip. In the control group (*n* = 10), no spectacle frame or lens was fixed. The experimental mice were housed in isolation, and the control mice were housed in groups of five. The body weight was measured weekly in experimental group from the time of purchase (P21) to the sacrifice date (P56). All mice were exposed to a 12 h light : 12 h dark cycle and checked daily for 28 days to assess the defocusing lens position stability. Then mice aged P56 were refracted and measured again to assess the effectiveness of myopia induction. In addition, a coronal segment of the tissue corresponding to the locations of the underlying screws was serially sectioned and H&E stained.

### 2.4. Ethics Statement

This study was approved by the Institutional Animal Care and Use Committee (IACUC) at Zhejiang University (Permit number: Zju201306-1-01-066). All experimental procedures were conducted in accordance with the Guidelines for the Care and Use of Laboratory Animals of Zhejiang University. All mice were sacrificed by an anesthetic overdose, and all efforts were made to minimize suffering.

## 3. Results

**Figure 5:**
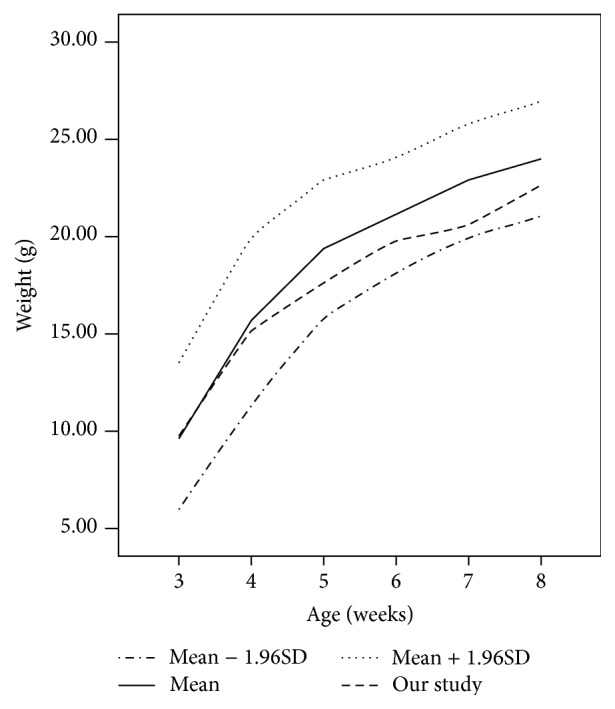
A body weight chart for the mice in experimental group. Reference values (mean, mean ± 1.96SD) were obtained from the JAX LAB (http://jaxmice.jax.org/support/weight/000664.html). The mean weight of mice in experimental group falls between the reference mean and mean − 1.96SD.

There were no intraoperative complications, and none of the animals experienced postoperative incision infection. The mice wearing head-mounted spectacle frames appeared to have normal activity and were well groomed and freely feeding (see video in Supplementary Material available online at http://dx.doi.org/10.1155/2016/8497278). Mice weights were monitored weekly, and weight gain of mice in experimental group was appropriate when compared with the reference values from the JAX LAB (Figure 5).

There were two situations to be noted in the assessment of device stability, a spectacle frame “lost” and an entire apparatus “lost.” In these cases, the defocusing lens could flip away from the eye and allowed for some normal visual input, which required timely intervention. The “lost” of spectacle frame was always the result of a loosened screw cap or nose pad and could be retightened in minutes. An entire “lost” apparatus was usually caused by frequent scratching, tissue reaction, or accidental clamping with the cage, and the nylon connector and spectacle frame should be refixated under general anesthesia. In this study (Table 1), 2 cases of spectacle frame loss but no entire apparatus loss were found in the first week (P29–P35), 8 cases of spectacle frame loss and 2 cases of entire apparatus loss occurred in the second week (P36–P42), 12 cases of spectacle frame loss and 3 cases of entire apparatus loss occurred in the third week (P43–P49), and 13 cases of spectacle frame loss and 3 cases of entire apparatus loss occurred in the fourth week (P50–P56). With this technique, 9/30 mice lost the spectacle frame or entire apparatus once, 14/30 mice lost the spectacle frame or entire apparatus twice, and 2/30 lost the spectacle frame or entire apparatus three times during the 28 days induction period. In the first two weeks, no mice lost the spectacle frame or entire apparatus three or more times. Our apparatus stability was relatively superior to the way that directly glued to the fur and was essentially the same as the head-mounted goggling apparatus reported by Faulkner et al. [18] (Table 2).

Refractive error data in Table 3 demonstrated no significant difference between the right and left eyes in the experimental group at P28 (0.08 ± 1.26 D; paired *t*-test *p* = 0.75) or in the controls (0.70 ± 1.34 D; paired *t*-test *p* = 0.13). 28 days later, the right eye of mice in experimental group showed a significant myopic shift compared to the left eye (−4.90 ± 2.17 D; paired *t*-test *p* = 1.69 × 10^−9^), but not found in the control group (−0.90 ± 1.37 D; paired *t*-test *p* = 0.07). The axial length measurements using OCT (Table 3) showed no significant difference between the right and left eyes in both experimental (0.004 ± 0.034 mm; paired *t*-test *p* = 0.58) and control group (0.004 ± 0.016 mm; paired *t*-test *p* = 0.48) at P28. After the induction period, the right eyes of mice in experimental group were significantly longer than the contralateral eyes (0.025 ± 0.037 mm; paired *t*-test *p* = 0.002), while no significant difference was found in the controls (−0.013 ± 0.031 mm; paired *t*-test *p* = 0.22).

**Figure 6:**
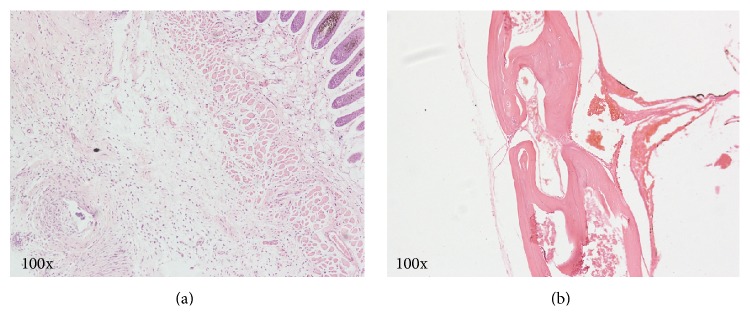
H&E stained sections of the skin (a) and the skull (b) at the screw sites. Note the reactive hyperplasia of the subcutaneous tissue, and the absence of a glaucomatous reaction or destructive changes to the skull.

Postmortem evaluation showed general reactive hyperplasia of the skin and no obvious alteration of the skull underlying the screws. Histological examination revealed chronic inflammation of the hypoderm due to the foreign body reaction in the H&E stained sections when compared with the normal controls, and the skull at the screw locations appeared healthy with no bony destruction (Figure 6).

## 4. Discussion

It is generally known that form deprivation and lens defocusing are the two classical methods for myopia induction in animal, and they were also reported to be used in the study of mouse myopia [9, 21]. No matter which method is chosen, existing techniques for device attachment can be shared. In this study, we used the lens-induced myopia model for the reason of renewed interest that myopia progression in humans and animals can be altered through optical intervention [5, 22].

A literature survey showed that Tejedor and de la Villa made the first attempts by lid-suturing to induce deprivation myopia in mouse; however, it was not suitable for defocusing lens attachment [15]. Later, Schaeffel et al. introduced the method that goggles directly glued to the fur around the eye, and it was improved by Barathi et al. to be used in lens-induced myopia [16, 17]. The method using glue was technically simple, but the device stability was not reliable enough and may lead to poor ocular health. Thus, Tkatchenko et al. reported a further refined technique, using a combination of glue and sutures [11]. Although the application of this method may not be restrained by mouse age and the effectiveness of myopia induction was reported to be good, no compliance data was shown. And our informal observations revealed that the stability has yet to be improved. Then some researchers used plastic collar to improve stability; however, it would inevitably affect the physical activity and whole health [11, 17]. Faulkner et al. developed a noncontact head-mounted goggle apparatus for the study of murine myopia [18]. It was reported to have satisfactory position stability and effective induction. However, the surgical procedures for the delicate device were quite complicated and time consuming and may lead to skull or brain injury. In addition, it was not suitable to be used in younger mouse with fragile skull, which may also make restriction on its popularity. Thus, it is still necessary to invent an easily operated, safe, and effective device for the myopia induction in mice.

Thus we developed the head-mounted spectacle frame and introduced a relatively easy-to-learn fixation method. Firstly, this device is lightweight (the nylon connector, spectacle frame, and defocusing lens amount to 0.50–0.60 grams) and could enhance the compliance of spectacle frame wearing. Comparing with the head-mounted goggle apparatus, our research results demonstrated at least evenly matched position stability. In our study, there were still situations that indicated that the defocusing lens was not permanently fixed, and the ability to maintain device stability tended to decrease with extended induction time. Therefore, it was of great importance to tighten the screws when operating and to check the fitting situation carefully during the experimental period. Secondly, our device showed satisfactory safety. The body weight gradually increased during the experimental period, with no obvious difference with the reference values. With sutures fixation but not skull implantation, there were only chronic subdermal inflammations found at the screw locations, but no pathological change in the skull or adjacent brain. Thirdly, and perhaps most importantly, our device and method induced myopic shift and axial elongation in the experimental eyes. Literatures have shown that a −10 D lens fitted over the mouse eye could induce a refractive shift of −13.03 D in 46 days (P10–P56) under a 12 : 12 h light : dark cycle [16], and a −25 lens induced a 14.6 D myopic shift in 21 days (P24–P45) under constant light [11]. In this study, the myopic shift of 4.90 D in 28 days (P29–P56) is relatively small and is approximately equal to that reported by Faulkner et al. using a head-mounted goggling apparatus (5.66 D myopic shift in 14 days of form deprivation) [18]. Many explanations may contribute to the reduced effectiveness, such as species variability of the mice, a later induction initiation, or others. In any case, the head-mounted spectacle frame apparatus is reliable and practicable. In addition, our device is economical and the surgical procedures are easy to learn, for only requiring commonly used microscopic suture materials but not professional equipment. This is also a beneficial aspect of our device and method in consideration of the popularization.

Some limitations should be considered in the current study. First, the physical dimensions of the one-piece frame were fixed in this study, which may not be compatible with mouse younger than P28. Thus frames with optimized physical dimensions adapting to younger mice during the susceptible period for myopia induction could substantially increase a future use of this device. Second, the introduced method is a rather invasive procedure, which requires recovery and infection and pain management. Thus design of inflammation and pain management may be necessary in further study. Third, although other reported methods and procedures were not easy to duplicate, it was still of significance to make comparison between these methods and our method at the same time, including effectiveness, security. And experiments along these lines are currently in progress in our laboratory.

In summary, we described a novel apparatus named the head-mounted spectacle frame apparatus and represented a simplification of the currently employed head fixation techniques for study of myopia in mouse. Our results demonstrated several advantages, including the improved compliance, effective myopia induction, and reliable security. The new method also has the potential to test form deprivation myopia. All of these features would likely prove useful in small animal models among investigators studying experimental myopia trials.

## Supplementary Material

Video recordings of the experimental mouse wearing the head-mounted spectacle frame.
a) Mouse has normal activity in breeding cage or out, without any restriction of limbs.
b) Mouse has active self grooming behavior in the cage.
c) Mouse has freely feeding behavior during the induction period.

## Figures and Tables

**Table 1:** Stability assessment of the head-mounted spectacle frame apparatus.

Experimental duration	# times spectacle frame was lost	# times entire apparatus was lost	# times defocusing lens was lost^a^
First week	2	0	2
Second week	8	2	10
Third week	12	3	15
Fourth week	13	3	16

Total	35	8	43

^a^Times defocusing lens was lost indicate the total of times spectacle frame was lost and times entire apparatus was lost.

**Table 2:** A comparison of the reported apparatus stability and the head-mounted spectacle frame.

Compliance (# times defocusing lens was lost)	≤2	≥3

Our apparatus	30/30	0/30

Head-mounted goggling apparatus [18]	24/28	4/28

Glued to the fur [18]	13/30	17/30

Our apparatus versus the head-mounted goggling apparatus, Fisher's Exact Test, *p* = 0.0482.

Our apparatus versus the glued to the fur technique, Fisher's Exact Test, *p* < 0.001.

**Table 3:** Results of retinoscopy refraction and axial length measurements.

	Age	Relative refraction (diopters)		Axial length (mm)	
		OD-OS	*p* value^*∗*^	OD-OS	*p* value^*∗*^
Experimental group	P28	0.08 ± 1.26	0.753	0.004 ± 0.034	0.577
	P56	−4.90 ± 2.17	1.69 × 10^−9^	0.025 ± 0.037	0.002

Control group	P28	0.70 ± 1.34	0.132	0.004 ± 0.016	0.482
	P56	−0.90 ± 1.37	0.068	−0.013 ± 0.031	0.220

Experimental group: *n* = 25; control group: *n* = 10.

OD-OS: values indicated the difference between the right and left eyes.

^*∗*^Student's paired *t*-test.
